# Influence of Comorbidities on Therapeutic Progression of Diabetes Treatment in Australian Veterans: A Cohort Study

**DOI:** 10.1371/journal.pone.0014024

**Published:** 2010-11-17

**Authors:** Agnes I. Vitry, Elizabeth E. Roughead, Adrian K. Preiss, Philip Ryan, Emmae N. Ramsay, Andrew L. Gilbert, Gillian E. Caughey, Sepehr Shakib, Adrian Esterman, Ying Zhang, Robyn A. McDermott

**Affiliations:** 1 Quality Use of Medicines and Pharmacy Research Centre, Sansom Institute for Health Research, University of South Australia, Adelaide, South Australia, Australia; 2 Discipline of Public Health, University of Adelaide, Adelaide, South Australia, Australia; 3 Department of Clinical Pharmacology, Royal Adelaide Hospital, Adelaide, South Australia, Australia; 4 School of Nursing and Midwifery, Sansom Institute for Health Research, University of South Australia, Adelaide, South Australia, Australia; 5 SA/NT Datalink, Sansom Institute for Health Research, University of South Australia, Adelaide, South Australia, Australia; University of Padova, Italy

## Abstract

**Background:**

This study assessed whether the number of comorbid conditions unrelated to diabetes was associated with a delay in therapeutic progression of diabetes treatment in Australian veterans.

**Methodology/Principal Findings:**

A retrospective cohort study was undertaken using data from the Australian Department of Veterans' Affairs (DVA) claims database between July 2000 and June 2008. The study included new users of metformin or sulfonylurea medicines. The outcome was the time to addition or switch to another antidiabetic treatment. The total number of comorbid conditions unrelated to diabetes was identified using the pharmaceutical-based comorbidity index, Rx-Risk-V. Competing risk regression analyses were conducted, with adjustments for a number of covariates that included age, gender, residential status, use of endocrinology service, number of hospitalisation episodes and adherence to diabetes medicines. Overall, 20134 veterans were included in the study. At one year, 23.5% of patients with diabetes had a second medicine added or had switched to another medicine, with 41.4% progressing by 4 years. The number of unrelated comorbidities was significantly associated with the time to addition of an antidiabetic medicine or switch to insulin (subhazard ratio [SHR] 0.87 [95% CI 0.84–0.91], P<0.001). Depression, cancer, chronic obstructive pulmonary disease, dementia, and Parkinson's disease were individually associated with a decreased likelihood of therapeutic progression. Age, residential status, number of hospitalisations and adherence to anti-diabetic medicines delayed therapeutic progression.

**Conclusions/Significance:**

Increasing numbers of unrelated conditions decreased the likelihood of therapeutic progression in veterans with diabetes. These results have implications for the development of quality measures, clinical guidelines and the construction of models of care for management of diabetes in elderly people with comorbidities.

## Introduction

Several studies have shown that glycemic control remains suboptimal in many patients with diabetes [1], [2], [3], [4], [5]. Progressive decline of β-cell function and consequent deterioration of glycemic control means that most patients eventually require treatment intensification with increased doses and/or the addition of other antidiabetic medicines. In a US study, intensification of diabetes treatment was lowest in the oldest patients and those with additional chronic diseases [6]. Appropriate care of elderly patients with comorbidities may involve choices that tailor intensity of diabetes treatment to individual patient characteristics such as life expectancy, cognitive impairment or patients' preferences [7].

The presence of competing demands due to comorbidity may also mean that the most pressing or symptomatic problems are prioritised. In 2006, Piette and Kerr proposed a framework for understanding diabetes care within the context of comorbid chronic conditions [8]. They hypothesized that comorbid conditions unrelated to diabetes (conditions that do not share the same pathogenesis or management plan as diabetes) may negatively influence the therapeutic management of diabetes. This has been demonstrated for conditions other than diabetes. The likelihood of treatment intensification of hypertension was shown to decrease with increasing numbers of unrelated comorbid conditions in US primary care practices [9]. Another study established that the number of unrelated conditions was negatively associated with guideline-based hyperlipidemia management in patients with hyperlipidemia, even in patients at the highest risk for cardiovascular events and cardiac death [10].

Within the population with type 2 diabetes, a small study involving 211 patient encounters assessed the effect of additional patient concerns on changes in antidiabetic medications [11]. No large population studies have been undertaken in patients with type 2 diabetes. The aim of this study was therefore to examine if the number of comorbid conditions unrelated to type 2 diabetes delayed therapeutic progression of diabetes treatment in Australian veterans.

## Materials and Methods

### Data sources and patient selection

We undertook a retrospective cohort study using the Australian Department of Veterans' Affairs (DVA) claims database. The DVA claims databases contain details of all prescription medicines, medical and allied health services and hospitalisations subsidised by DVA. The study period was from 1 July 2000 to 30 June 2008. Veterans were included if they were eligible for all health services subsidised by the DVA in the 12 months prior to the date of their first (index) type 2 diabetes prescription. The study included new users of metformin or sulfonylurea medicines defined as those with at least two successive prescriptions of the same medicine and no dispensings of these medicines in the 12 months prior to the index prescription. Users of other classes of antidiabetic medicines (insulin, other oral medications, combinations of metformin) were excluded as prescription of these medicines at the initiation of diabetes treatment is uncommon in Australia and may relate to unusual clinical circumstances [12].

### Outcome definitions

Two outcomes were assessed: time to addition of a new oral antidiabetic treatment (metformin, sulfonylura, acarbose or thioglitazone) or switch to insulin, and time to addition of a new antidiabetic treatment or switch to any other antidiabetic treatment. As the database does not include information on the prescribed dose or duration of treatment, the duration of use of prescriptions was defined as the number of days within which 75% of people refilled their prescriptions, as calculated from the dataset [13].

Comorbid conditions unrelated to diabetes were identified by using prescription dispensed data from 1 July 1999, based on categories of the Rx-Risk-V model, a pharmaceutical-based co-morbidity index which has been validated in the Australian veteran population [14] (Appendix S1). Some chronic conditions are unlikely to subside in the long term and were considered permanently present from the first dispensing of the corresponding prescription until the end of the follow-up. Other conditions were considered present as long as the corresponding prescription was dispensed. The sum of all unrelated comorbid conditions was updated on a daily basis up to the index anti-diabetic prescription. Seven unrelated comorbid conditions common in the elderly population were also determined from hospital discharge diagnosis (ICD-10) and/or ATC codes measured between the index prescription and the outcome or study end (Table 1).

**Table 1:** Codes used to define specific conditions.

Diseases	Selection criteria
Cancers, except non-malignant skin cancer	ICD-10 codes: C00-C97 (excluding C44), at least one hospitalisation in the follow-up period
Chronic obstructive pulmonary disease	ICD-10 codes: J44 or taking inhaled anticholinergic drugs (ATC code: R03BB*), at least one dispensing in the follow-up period
Dementia	Dispensed anticholinesterases (ATC code: N06DA)
Depression	Dispensed non-tricyclic antidepressants (ATC codes: N06AB, N06AG02, N06AX no substring), dispensings covering at least 60% of the follow-up period
Urinary incontinence	Urinary antispasmodics (ATC code GO4BD), at least one dispensing in the follow-up period
Parkinson's disease	Antiparkinson agents (ATC codes N04AA01-N04BX02, at least one dispensing in the follow-up period

### Statistical analysis

In this elderly veteran population, death is a competing risk event which may preclude the onset of the outcome of interest, therapeutic progression. For this reason, we calculated the cumulative incidence of outcome of interest in the presence of competing risk events. Competing risk regression analyses were conducted using the Fine and Gray approach which extends the Cox model to competing risks data by considering the sub-distribution hazard [15]. The strength of the association between each variable and the outcome was assessed using the sub-hazard ratio, which is the ratio of hazards associated with the cumulative incidence function under different values of the covariates. Covariates included age at entry, gender, residential status (community dwelling or age-care resident), having used an endocrinology service, number of hospitalisation episodes (between the index diabetes prescription and therapeutic progression or end of the study), and adherence to diabetes medicines (defined as sufficient medicine dispensed to cover 80% to 120% of the treatment duration). Residential status, use of an endocrinology service and adherence were time varying and adjusted each year. Data rendering was performed using SAS 9.1 and statistical analyses were performed using Stata 11.0.

## Results

**Figure 1:**
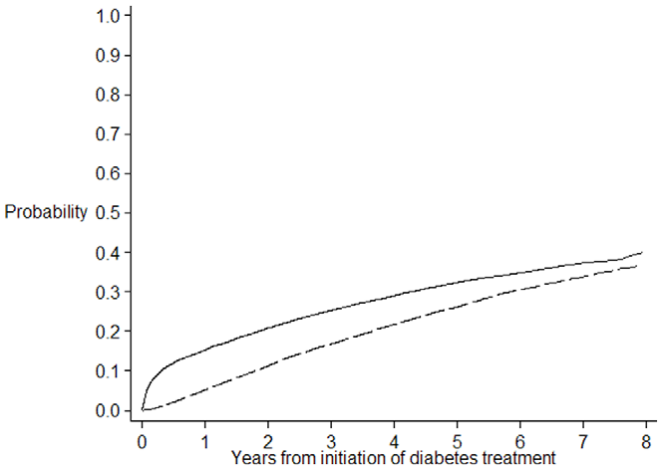
Cumulative incidence of therapeutic progression by addition of an antidiabetic treatment or switch to insulin. The plain line represents the cumulative incidence of therapeutic progression by addition of an antidiabetic treatment or switch to insulin. The dash line represents the cumulative incidence of the competing risk event, death, occurring prior to therapeutic progression.

**Figure 2:**
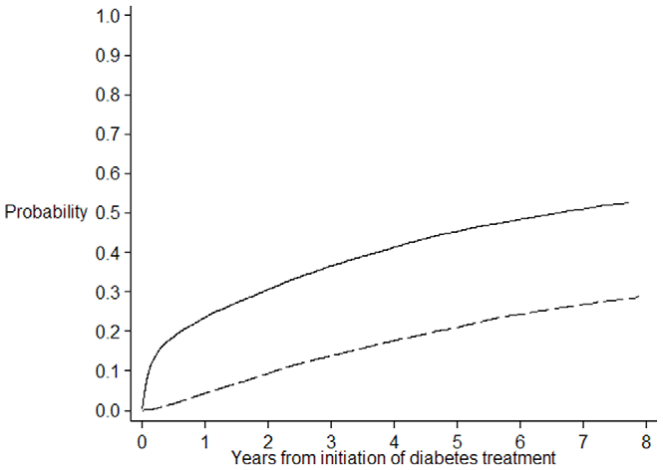
Cumulative incidence of therapeutic progression by addition of an antidiabetic treatment or switch to any antidiabetic medicine. The plain line represents the cumulative incidence of therapeutic progression by addition of an antidiabetic treatment or switch to any antidiabetic medicine. The dash line represents the cumulative incidence of the competing risk event, death, occurring prior to therapeutic progression.

Overall, 20134 veterans were included in the study with a mean age of 77.3 years (SD 9.1), 64% were men, 58% were dispensed metformin and 42% sulfonylureas. The cumulative incidences of therapeutic progression by addition or switch to another antidiabetic medicine are presented in Table 2 and shown in Figures 1 and 2. At one year, 23.5% of patients with diabetes had a second medicine added or had switched to another diabetes medicine, 41.4% at 4 years and 51.0% at 7 years.

**Table 2:** Cumulative incidence of therapeutic progression.

	Addition of an antidiabetic medicine or switch to insulin			Addition of an antidiabetic medicine or switch to any antidiabetic medicine		
	1 year%	4 years%	7 years%	1 year%	4 years%s	7 years%
Cumulative incidence of therapeutic progression	15.3	29.0	37.4	23.5	41.4	51.0
95% CI	14.8–15.8	28.3–29.7	36.5–38.4	22.9–24.1	40.6–42.1	50.1–51.9
Cumulative incidence of death	5.2	21.7	33.9	4.3	17.7	26.9
95% CI	4.9–5.6	21.1–22.5	33.0–34.9	4.0–4.6	17.1–18.3	26.0–27.7

The results of the competing risks multivariable regression analyses for the number of unrelated comorbidities and specific geriatric conditions are shown in Table 3. The number of unrelated comorbidities was significantly associated with the time to addition of an antidiabetic medicine or switch to insulin (subhazard ratio [SHR] 0.87 [95% CI 0.84–0.91], P<0.001). Depression, cancer, chronic obstructive pulmonary disease, dementia, and Parkinson's disease, were significantly associated with a decreased likelihood of therapeutic progression. The covariates age, residential status, number of hospitalisations and adherence to antidiabetic medicines were all negatively associated with therapeutic progression. The use of endocrine services increased the likelihood of therapeutic progression

**Table 3:** Competing risk proportional hazard regression analyses of time to therapeutic progression.

	Addition of an antidiabetic medicine or switch to insulin					Addition of an antidiabetic medicine or switch to any antidiabetic medicine						
	SHR	95% CI		P value		SHR		95% CI		P value		
Number of unrelated comorbidities	0.872	0.839–0.905		<0.001		0.879		0.852–0.907		<0.001		
Cancer	0.703		0.590–0.836		<0.001		0.690		0.605–0.788		<0.001	
Chronic obstructive pulmonary disease	0.866		0.778–0.963		0.008		0.855		0.786–0.930		<0.001	
Dementia	0.682		0.519–0.895		0.006		0.636		0.512–0.791		<0.001	
Depression	0.803		0.742–0.868		<0.001		0.781		0.734–0.832		<0.001	
Urinary incontinence	0.794		0.542–1.164		0.237		0.752		0.555–1.02		0.067	
Parkinson's disease	0.565		0.457–0.698		<0.001		0.635		0.544–0.742		<0.001	
Age	0.975	0.972–0.977		<0.001		0.981		0.978–0.983		<0.001		
Number of hospitalisations	0.776	0.744–0.809		<0.001		0.811		0.788–0.834		<0.001		
Adherence to antidiabetic medicines	0.599	0.556–0.644		<0.001		0.545		0.513–0.579		<0.001		
Residency (aged care versus community)	0.892	0.853–0.934		<0.001		0.884		0.849–0.920		<0.001		
Endocrinology service	1.196	1.145–1.250		<0.001		1.317		1.269–1.367		<0.001		

SHR, Subdistribution Hazard Ratio from multivariable competing risk regression analyses.

## Discussion

The results of this large population based study demonstrated that one year after initiation of diabetic therapy, 23.5% of patients had a second medicine anti-diabetic medicine added or had switched to another medicine. Forty-one percent progressed after 4 years. Further, increasing numbers of unrelated comorbid conditions was found to be associated with a decreased likelihood of therapeutic progression.

Whilst most previous studies examining therapeutic progression have only followed patients for one year, and, in general, included younger participants than in our study, the current results were however similar. In a Dutch study of more than 46,000 new users of antidiabetic medicines, the proportion of patients with an addition or switch of antidiabetic treatment at 1 year ranged from 20.2% with a sulfonylurea initial treatment to 25.7% with metformin initial treatment [16]. In two earlier American studies involving 85,888 members of an American managed care organization and 19,900 members of a pharmacy benefit management organization, rates of therapeutic progression at 1 year were 24.5% [17] and 17.2% respectively [18]. In 4556 members of a US health care organization receiving a sulfonylurea, the time to addition or switching to a second antidiabetic medicine at 4 years ranged between 40% and 70% depending on the level of glycated hemoglobin achieved during the first year of treatment [19].

We confirmed our main hypothesis, that more unrelated conditions decreased the likelihood of treatment intensification. This result is consistent with a study involving 211 patient encounters which showed that, among patients with a glycated hemoglobin level greater than 7%, each additional patient concern was associated with a 49% reduction in the likelihood of a change in medication, independent of length of the encounter and most recent glycated hemoglobin level [11]. The lower likelihood of treatment intensification in elderly people with more unrelated conditions observed in our study is also consistent with clinical guidelines on treatment of diabetes which recommend a higher target glycated haemoglobin may be appropriate in the elderly population or patients with comorbidities [20]. While collectively, unrelated comorbidity was associated with less likelihood of treatment intensification, the presence of depression, cancer, chronic obstructive pulmonary disease, dementia, or Parkinson's disease, specifically, were associated with a decreased likelihood of treatment intensification. Patients were also less likely to have their antidiabetic treatment intensified if they were older, adherent to their antidiabetic medicines, living in aged care, or had been hospitalized in the prior year. Patients were more likely to have their antidiabetic treatment intensified if they had visited an endocrinologist.

The DVA database does not include data on glycated hemoglobin and we could not determine the rates of true therapeutic failure (the inability to achieve metabolic control when a treatment is first initiated) nor adjust for this variable in the statistical analysis. Treatment intensification per se cannot be taken to represent good diabetes management and it may expose patients to increased risk of adverse effects. However, treatment intensification has been shown to be strongly associated with improved control of diabetes [21], [22] and may represent a reliable surrogate measure of the quality of clinical care. In addition, some forms of treatment intensification such as increasing the dose of an antidiabetic medicine could not be measured in the DVA data.

Piette's framework on the impact of comorbid chronic conditions on diabetes care is useful for understanding how elderly people with multiple conditions are managed in clinical practice [8]. Health professionals and patients face multiple and sometimes conflicting challenges in deciding on the best health care options. Intensive glycemic control may reduce risk of diabetes microvascular complications but its effect on cardiovascular outcomes is more controversial. A decision analysis showed that, among older patients with diabetes, the presence of multiple comorbid illnesses or functional impairments was an important predictor of diminishing expected benefits of intensive glycemic control [23]. These results were confirmed in a recent study that showed that patients with high levels of comorbidity received diminished cardiovascular benefit from intensive glycemic control [24]. Ideally, a new multimorbidity framework is required that would overcome the limited perspective of single disease focused models of care, and would aid in prioritizing treatments according to expected benefits over time, potential harms, comorbid conditions, disabilities and patients' preferences. It would also seek to inform the multiple trade-offs associated with the management of multiple comorbidities [25].

Our study was performed in the Australian veteran population. Although the results may not be able to be generalized to the whole elderly Australian population, the use of health services by veterans has been shown to be similar with the rest of the Australian community after adjustment for disability [26]. With the current development of important healthcare reforms in Australia, our results may inform future construction of performance management measures for diabetes management that includes the consideration of elderly patients with multiple conditions.

## Supporting Information

Appendix S1 (0.05 MB DOC)
