# Supplementary material for: Influence of Comorbidities on Therapeutic Progression of Diabetes Treatment in Australian Veterans: A Cohort Study
Source: PLoS One. 2010 Nov 17;5(11):e14024. doi: 10.1371/journal.pone.0014024 (PMC2984440; doi:10.1371/journal.pone.0014024)
Supplement: Appendix S1 — (0.05 MB DOC) [file pone.0014024.s001.doc]

**Table 1. ATC codes used** for unrelated comorbid conditions

| **Unrelated comorbid conditions** | **Medicines** | **ATC codes** | **Duration of condition** |
| --- | --- | --- | --- |
| Alcohol dependence | Acamprosate, naltrexone | N07BB03 - N07BB04, V03AA01 | Indefinite |
| Allergies | Antihistamines (except hydroxyzine and diphenhydramine), nasal antiinflammatories | R01AC01 - R01AD60, R06AD02 -R06AX26 | As calculated in the dataset* |
| Anxiety | Anxiolytics (benzodiazepines) | N05BA01-N05BA12 | As calculated in the dataset* |
| Benign prostatic hypertrophy | Alpha blockers | G04CA02-G04CA03, G04CB01 | As calculated in the dataset* |
| Bipolar disorder | Lithium | N06AX – no substring | Indefinite |
| Dementia | Donepezil, tacrine | N06DA02-N06DA04 | Indefinite |
| Depression | Antidepressants | N06AA01-N06AG02, N06AX03-N06AX18 | As calculated in the dataset* |
| End stage renal disease | Alpha erythropoietin, calciferol, calcitriol, sevelamer | B03XA01-B03XA02, A11CC01-A11CC04,V03AE02 | Indefinite |
| Epilepsy | Anticonvulsants | N03AA01-N03AX14 | As calculated in the dataset* |
| Gastric acid disorder |  | H2RA, PPI A02BA01-A02BX05 | As calculated in the dataset* |
| Glaucoma | Topical antiglaucoma agents | S01EA01-S01EB03,S01EC03-S01EX | Indefinite* |
| Gout | Antigout agents | M04AA01-M04AC01 | As calculated in the dataset* |
| Hepatitis C | Interferon/ribavirin combinations | J05AB54 | Indefinite |
| HIV | Anti-HIV antivirals | J05AEJ05AE08, J05AF01-J05AG03, J05AR, J05AX07 | Indefinite |
| Hypothyroidism | Thyroid replacements | H03AA01-H03AA02 | Indefinite |
| Inflammatory bowel syndrome | IBS specific drugs, rectal antiinflammatories | A07EC01-A07EC04, A07EA01-A07EA02 | Indefinite |
| Liver failure | Lactulose | A06AD11 | Indefinite |
| Malignancies | Antineoplastics agents (excluding topical) | L01AA01-L01XX31 | Indefinite |
| Migraine | Antimigraine medications | N02CA01-N02CX01 | Indefinite as long as one dispensing over each 6-month study period |
| Osteoporosis/Paget’s disease | Alendronate, etidronate | M05BA01-M05BB03 | Indefinite |
| Pain | Opiate containing medications | N02AA01-N02AX02 | As calculated in the dataset* |
| Pain/inflammation | NSAIDS | M01AB01-M01AH06 | As calculated in the dataset* |
| Pancreatic insufficiency | Pancreatic exocrine enzyme replacement | A09AA02 | As calculated in the dataset* |
| Parkinson’s disease | Antiparkinson agents | N04AA01-N04BX02 | Indefinite |
| Psoriasis | Systemic and topical antipsoriatics | D05AA,D05BB01-D05BB02, D05AX02 | Indefinite |
| Psychotic illness | Antipsychotics | N05AA01-N05AB02, N05AB06-N05AX12 | Indefinite |
| Reactive airways diseases | Inhaled bronchodilators | R03AC02-R03DC03 | As calculated in the dataset* |
| Smoking cessation | Nicotine, bupropion | N07BA01-N07BA02 | As calculated in the dataset* |
| Steroid-responsive conditions | Glucocorticoids | H02AB01-H02AB10 | Indefinite as long as one dispensing over each 6-month study period |
| Transplant | Immunosuppressants | L04AA01-L04AA21 | Indefinite |
| Tuberculosis | Anti-tubercular agents | J04AB04-J04AK02 | As calculated in the dataset* |
| Urinary incontinence | Urinary antispasmodics | ATC GO4BD | Indefinite |

***** The condition was considered present as long as the corresponding prescription was used, with the duration of use defined as the number of days in which 75% of people returned for a prescription refill as calculated in the dataset.
